# Immunotherapeutic Activities of a DNA Plasmid Carrying the Mycobacterial hsp65 Gene (DNAhsp65)

**DOI:** 10.3389/fmedt.2020.603690

**Published:** 2020-12-15

**Authors:** Celio Lopes Silva, Thiago Malardo, Aline Seiko Carvalho Tahyra

**Affiliations:** ^1^Department of Biochemistry and Immunology, Medical School of Ribeirão Preto, University of São Paulo, Ribeirão Preto, Brazil; ^2^Farmacore Biotecnologia Ltda, Ribeirão Preto, Brazil

**Keywords:** DNAhsp65, heat-shock protein, DNA vaccine, infectious diseases, allergy, cancer, autoimmune diseases, immunotherapy

## Abstract

DNA vaccines have become relevant subject matter, and efforts for their development have been increasing due to their potential as technology platforms applicable for prophylactic and therapeutic approaches for infectious diseases and for cancer treatment, allergies, and autoimmune diseases. This review aimed to summarize current knowledge about the plasmid DNA vaccine carrying the mycobacterial hsp65 gene (DNAhsp65), which demonstrates immunomodulatory and immunoregulatory properties of both the innate and adaptive immune systems. The possible mechanisms associated with the modulation and regulatory role of DNAhsp65 in the control of various conditions is also discussed.

## Introduction

Vaccine development dates back to the 1790s and Edward Jenner, the pioneer of the smallpox vaccine (1). After that spark, several vaccines were subsequently developed based on attenuated, inactivated, and killed pathogens or vaccines composed of pathogen subunits. Notwithstanding, by 1980, a new chapter began in the history of vaccine development when Paoletti et al. (2) created a new approach to vaccination: the first use of recombined DNA as a vaccine development strategy. Such an approach opened doors for the development of the first DNA vaccines.

The principle of the method is based on the direct introduction of the plasmid into the tissue, allowing the expression of the target antigen and, consequently, the induction of an antigen-specific immune response (3). Plasmids are circular DNA vectors composed of a promoter region that drives transcription of the target gene, a multiple cloning site to insert genes of antigenic interest, an origin of replication site, and an antigenic resistance gene (3). The DNA vaccination strategy has gained the spotlight due to the ease of production, the multiple forms of administration, the molecular stability, the biological safety, and the protective immune response (3).

The DNA vaccine developed by our research group consists of a plasmid construction containing the 3,144 base pair (bp) heat shock protein 65 (HSP65) gene of *Mycobacterium leprae* inserted into the pVAX1 vector flanked by BamHI and NotI restriction sites. The plasmid construct, named DNAhsp65, also encodes the cytomegalovirus (pCMV) promoter sequence, the bovine growth hormone (BGH pA) polyadenylation sequence, the origin of pUC vector replication (pUC ori), the kanamycin resistance gene, and the T7 promoter-priming site. The physicochemical features of the recombinant plasmid and the end product, the hsp65 protein, were characterized by several assays in both cell culture and animal models for standardization. Interestingly, the DNAhsp65 vaccine showed effective capacity for controlling infectious diseases, autoimmune diseases, allergy, and tumors. The ability to act under such a wide variety of immunopathologies is associated with the immunomodulatory potential and with inherent features of the structure and composition of DNAhsp65 that will be highlighted (4).

Among a range of potential immunobiological compounds capable of controlling and regulating the immune system, heat shock proteins (HSPs) have gained focus since their discovery and the observation that immune responses to HSPs readily developed under various pathological conditions (5–12). With respect to their evolutionary conservation over diverse species, HSPs' roles in maintaining the integrity of cellular proteins and their stress inducibility, as well as their potential impact on the organization of immune reactivity in mammals, are considered to be broad and multifaceted (5). Their influence in pathways of the innate and adaptive immune systems has been described (5), highlighting their intracellular role in antigen presentation, their expression of innate receptors, and their extracellular role in tumor immunosurveillance and autoimmunity.

Exogenous administration of HSPs has been used in experimental models of immunotherapy for cancer, infectious diseases, allergy, and autoimmune diseases to elicit immunotherapeutic responses. Among the several classes of these molecules, the most important from the point of view of immunomodulatory properties are hsp70, hsp96, and hsp60 (5). Mycobacterial HSPs, primarily Hsp65 and Hsp70, are known to modulate both the innate and adaptive (cellular and humoral) aspects of the immune system (7, 13).

The primal functions of HSPs began to acquire a new facet by 1980 when it was demonstrated that hsp70 isolated from tumor cells induced immunity to cancers, whereas the corresponding preparation of protein derived from normal tissue was unable to elicit protective immunity (6, 7). Subsequently, it was shown that this phenomenon is due to the chaperon function (loader) of HSPs that form complexes with antigenic peptides from tumor cells or from intracellular infectious agents (5).

The mechanisms by which the HSP-peptide complex is formed and how it stimulates and/or modulates the innate and adaptive immune responses have been reviewed in detail by many authors (5–12). Different levels of analyses were performed on the interaction of HSPs with peptides, taking into account the structure, biochemical features, and the immune relevance of these interactions (5). By 1996, the crystallographic structure of the bacterial DnaK protein, a homolog of mammalian hsp70, and its bound peptide were elucidated, and the peptide-binding groove for calreticulin, hsp90, and gp96 was subsequently identified, noting that the groove is unique for each HSP (5).

Antigenic peptides derive from a viral or bacterial infected cell, from tumor cells, or from major histocompatibility complex (MHC)-mismatched cells, reflecting the cellular antigenic signature. They are usually composed of 10–30 amino acids and originate from normal protein metabolism, which, in a determined fashion, become associated with cytosolic or endoplasmic reticulum HSPs that elicit cellular and humoral responses (5). Similar to the MHC molecule family, HSPs have come to light as one of the relevant clusters of peptide-binding proteins (7).

In the context of cancer, these complexes efficiently contributed to tumoral immunosurveillance by eliciting a specific immune response due to HSPs' adjuvant properties. Furthermore, these complexes have been isolated from tumors and applied as cancer immunotherapeutic interventions in human clinical trials, demonstrated using DNAhsp65 as a key for improving the antitumoral immune response (14).

Taking into account an infectious condition, HSPs' ability to complex with a wide array of microbial peptides, as well as to bacterial DNA and to lipopolysaccharide (LPS), suggests the possibility of improving the immune response (9). Along those lines, it was demonstrated, for the first time, that recombinant mycobacterial hsp65 can bind a wide variety of peptides and exhibits proteolytic activity toward both proteins and polypeptides (15). This activity was characterized using a fluorometric assay and by identifying fragments generated from certain polypeptides used as substrates. Two putative threonine catalytic groups of the mycobacterial hsp65 were identified from the alignment of its amino acid sequence with the heat shock protease HslVU of *Escherichia coli*. This hypothesis was strongly supported by site-directed mutagenesis studies of amino acid residues integrating the putative catalytic group, one of which led to the complete loss of the proteolytic activity of the mycobacterial hsp65 (15).

Moreover, *in silico* prediction of the tertiary structure of *M. leprae* hsp65 protein shows an unusual structure in the carboxy-terminal region (16). The results clearly opened up new approaches for the contribution of this stress protein in a range of pathophysiological processes. This class of bacterial protein, besides representing important mycobacterial antigens, should be regarded as a peptidase, which is able to generate or destroy other biologically active molecules possibly involved in these processes (15). This may have a critical implication for the DNAhsp65 mechanism of action. In summary, endogenous expression of hsp65 in tumor or infected cells acts by degrading antigenic proteins. The resulting peptides are complexed to hsp65 and stimulate specific immunity against these tumoral or microbial antigens.

## Innate and Adaptive Immune Responses Elicited By DNAHSP65

DNAhsp65 actively participates in the activation of innate immunity, acting as an endogenous adjuvant and playing a fundamental role in the activation and control of adaptive immunity (17–20). The immune response basically consists of a set of highly integrated reactions involved in the two-fold function of (i) recognition and (ii) elimination of foreign substances. The innate immune response constitutes the first line of host defense by which many potentially harmful exogenous and/or endogenous invaders are rapidly controlled or eliminated. The hallmark of innate immunity lies in its immediacy and swiftness of response that allows the system to react quickly during infections, and although it does not display the specificity and complexity of the adaptive immune response, it exhibits flexibility and adaptability against most foreign intruders using a defined group of receptors.

In addition, the innate response actively participates in and is required for the induction of adaptive immunity, which is responsible for inducing a specific response against foreign antigen presented by innate immune cells. Macrophages and dendritic cells (DCs) play a substantial role and are also known as antigen presenting cells (APCs). These cells recognize pathogen-associated molecular pattern (PAMP) *via* special receptors known as pattern recognition receptors (PRRs). Activation of PRRs leads to the release of immune mediators by macrophages and DCs in addition to the phagocytosis of infected cells. Experimental assays of activation of the innate immune system by DNAhp65 were performed to assess intracellular trafficking, transfection capacity, gene expression, cellular activation, release of immunological mediators, and activation molecules in the J774 macrophage cell line (21).

The potential of DNAhsp65 to stimulate human immune response in APCs has also been verified (17, 18). Franco and colleagues demonstrated that after 4 h of stimulation in culture, both macrophages (CD11b^+^/CD86^+^/HLA-DR^+^) and DCs (CD11c^+^/CD86^+^/CD123^−^/BDCA-4^+^/TFN-α-) were able to uptake the plasmid DNA (17). Both cell populations expressed mRNA for the hsp65 protein in addition to exhibiting high constitutive expression of Toll-like receptor 9 (TLR9), which is critical for cell–plasmid interactions through the unmethylated CpG site. After 48 h of stimulation, DCs exhibited positive regulation of costimulatory CD80 and CD86 molecules and produced significant concentrations of interleukin (IL)-12 (17). In contrast, macrophages did not show changes in their cellular phenotype, nor did they secrete IL-12. However, macrophages produced significant concentrations of IL-6, tumor necrosis factor (TNF)-α, and IL-10 (17) and exhibited a higher bactericidal capacity than DC cells, being more efficient in restricting the growth of microorganisms in assays following *in vitro* cellular infection with *Mycobacterium tuberculosis* (Mtb) (17).

In terms of the adaptive immune response, Wowk et al. (18) investigated whether DNAhsp65 or the recombinant Hsp65 protein (rHsp65) modulated activation and cytokine release from T lymphocytes (18). *In vitro* experiments were performed to mimic the prophylactic or therapeutic role of the Hsp65 antigen in cell culture from healthy donors and tuberculosis (TB) patients. Results suggested that T-CD4^+^ proliferation from the healthy group was stimulated by both the DNA vector and the recombinant protein, whereas T-CD8^+^ proliferation from healthy and tuberculous patients was observed only in response to rHsp65 stimuli. An increase in the frequency of IL-10^+^ cell populations of T-CD4^+^ and T-CD8^+^ was also observed from healthy individuals and from TB patients stimulated with DNAhsp65, indicating the immunoregulatory role of Hsp65 antigen in human cells (18).

Recently, the probable receptor for HSPs in DCs has been described in the literature (22, 23), despite some controversies existing on the matter (24–26). HSP reportedly interacts with Toll-like receptor 4 (TLR4) in DCs, triggering the activation of a signaling cascade involving TIR-domain-containing adapter-inducing interferon-β (TRIF) and myeloid differentiation primary response 88 (MyD88) adaptor proteins (22). These factors play a critical role in the activation of DCs induced by HSP. Likewise, this interaction among HSP-TLR4-TRIF-MyD88 is essential for the optimal activation and response of T-CD8^+^ lymphocytes (23).

Despite clear activation of the innate and adaptive immune systems by HSPs, denoting their high immunostimulatory potential, the response can be improved using adjuvants constituted by many different compounds. For instance, emulsions in the veterinary area or aluminum salts in human cases, with microspheres and liposome formulations as adjuvant-delivery systems indeed showing a significant enhancement of the efficacy of the vaccine in response to challenge with *M. tuberculosis* in a murine model (27, 28).

In view of this spectrum of results indicating the activation of innate and adaptive immunity and the interaction between them, it is relevant to emphasize that the plasticity of HSP-mediated immunity is a result of the ability of the protein to engage with costimulatory molecules of APCs and activate these cells. The outcome of the activation of multiple signaling cascades by different costimulatory and signaling receptors on APCs determines the subtype of T helper immune response.

## Prophylactic and Therapeutic Effects of DNAHSP65 Against Infectious Diseases

### Tuberculosis

Despite the worldwide incidence of TB decreasing by 2% per year, it remains as one of the main causes of death, especially in developing countries where it is considered a serious public health problem. According to data from the WHO Global Tuberculosis Report 2019, ~1.5 million people died of TB, and more than 10 million new cases were registered in 2018. This is primarily attributed to an insufficient immune response toward the bacillus.

Although the Bacillus Calmette-Guérin (BCG) vaccine is widely used, it has major limitations as a preventative measure. Effective treatment requires that patients take large doses of antibacterial drug combinations for at least 6 months after diagnosis, which is difficult to achieve in many parts of the world and is further restricted by the emergence of multidrug-resistant strains of *M. tuberculosis* (MDR-TB). Moreover, one-third of the world's population is estimated to have latent TB infection (LTBI) wherein they do not have active TB disease but may develop it in the near or remote future, a process known as “TB reactivation.”

The lifetime risk of reactivation for a person with documented LTBI is estimated to be 5–10%, with the majority developing TB disease within the first 5 years after the initial infection. However, the risk is considerably higher in the presence of predisposing factors, such as coinfection with HIV, helminths, and immunosuppressive treatments. This widely contributes to the high rates of incidence and prevalence of TB, since at any time a factor that causes immunosuppression can cause illness reactivation.

The primary strategies for effective TB control are related to the development of new preventive and/or therapeutic vaccines that will also be effective for the control of chronic TB and LTBI, preventing reactivation, potentiating the action of conventional treatments with antibacterial drugs, and assisting in the control of MDR-TB.

The first DNA vaccine for the prevention and treatment of TB was described by Lowrie et al. (29). Since then, the subject has been eagerly explored, primarily with a view to understand the immunogenic and protective potential of DNAhsp65 under challenge with Mtb in murine (29–36) and guinea pig (37) models.

For this purpose, the main subpopulations of cells that confer long-term immunity against TB were evaluated in experiments with mice immunized with both BCG and DNAhsp65 and subsequently infected with Mtb (38). The main subpopulation linked to the protective role of DNAhsp65 was CD8^+^CD44^hi^IFN-γ^+^, which was prominent even 8 or 15 months after vaccine administration. On the other hand, in response to BCG vaccination, the primary enriched subpopulation of cells was composed of CD4^+^CD44^lo^IFN-γ^+^.

To better comprehend the contribution of these different T lymphocyte populations, flow cytometry analysis was performed by gating cells into CD4^+^CD8^−^ and CD8^+^CD4^−^ and then into CD44^hi^ and CD44^lo^ populations. It was clearly evidenced that the CD44^lo^ cells were unable to provide protection in the adoptive lymphocyte transfer experiments. Only CD44^hi^ cells conferred a protective response, with an emphasis on CD8^+^CD4^−^ cells from the DNAhsp65 vaccinated group, which were demonstrated to be the most protective compared with BCG vaccinated animals (38).

In addition, after BCG or DNAhsp65 vaccination, the levels of protection conferred by T lymphocytes lasted 8 months and decreased after 15 months of vaccination (38). A range of other studies were conducted to evaluate different experimental models to further characterize the aspects of immunogenicity and vaccine efficacy of DNAhsp65 as a preventive method for TB (29–36).

After inhaling droplets containing viable bacillus, the infection begins with phagocytosis of the bacteria in the lower airways by resident alveolar macrophages. However, the ability of Mtb to hide inside these cells and evade the immune system triggers a latency process. It is well known that the fine-tuned interactions between the innate and adaptive responses, mediated primarily by macrophages, DCs and lymphocytes, are critical for activation of an effective immune response against Mtb. Therefore, the critical issue for immunologists is to identify the primary mechanism that allows T lymphocytes to play their important immunoprotective role (39).

Diving into this challenge, we began to describe and identify key points of the immune response during Mtb infection in mice and observed an abundance of IL-4-producing T helper lymphocytes, a signature of a type 2 response. Since it is well established that the prevalence of the type 2 response does not provide protection against Mtb infection, it was hypothesized that a balance toward the type 1 response, with a prevalence of cytotoxic T lymphocytes interferon (IFN)-γ^+^, would be beneficial (39).

Next, whether the BCG vaccine or DNAhsp65 would be able to produce this type 1 response before and during TB infection was investigated. Intriguingly, this phenotype of immunological response is inherent to DNAhsp65 and does not occur by preventive or therapeutic administration of BCG. Compared with BCG, the DNAhsp65 vaccine leads to a significant increase in the frequency of IFN-γ-producing cells that remains elevated even after challenge with Mtb, in contrast with results obtained from the group vaccinated with BCG.

In this group, after BCG administration, a subtle and equal increase in IFN-γ- and IL-4-producing lymphocytes is observed at the same level compared in animals only infected with Mtb and animals after preventive administration of BCG followed by challenge. Plainly, the optimistic results obtained from these experiments brought to light the possible application of DNAhsp65 as an immunotherapeutic complement to the chemotherapy treatment of TB (39).

In agreement with the immunotherapeutic use of DNAhsp65, one of the most relevant works published revealed that this plasmid vaccine operates as an immunomodulator against a well-established infection. This promising potential to treat chronic TB diseases, latent cases, and MDR-TB was published in 1999 (40) and in 2000 (41). It has been found that in animals with a high bacterial burden, DNAhsp65 immunotherapy led the immune response to switch from one that is relatively inefficient allowing bacterial stasis (Th2) to one that kills the bacteria (Th1), eliminating the persistent bacillus (40, 41). Another interesting data obtained by these investigations is that this immunotherapy might be a valuable tool to shorten the duration of antibacterial treatment (20, 39) in addition to enhancing the response against latent TB and MDR-TB (39, 40). The results also suggest that DNAhsp65 is able to prevent TB reactivation in immunosuppressed animals (39, 40) and could be administered in helminth coinfection cases (42–44).

The regulation of Th1, Th2, and Th17 subpopulations of T lymphocytes was also analyzed in TB in response to DNAhsp65 treatment (45). Since the Th1 profile antigen-specific response triggered by DNAhsp65 immunotherapy was effective against TB, it would be of great value to determine the activity profile of other pro-inflammatory pathways to avoid the immunopathology of the disease. Therefore, the cytokine response of IFN-γ and IL-17 was analyzed in parallel with the behavior of CD4^+^, CD8^+^, and γδT cells in the pulmonary environment of mice submitted to the immunotherapeutic treatment with DNAhsp65 after the challenge (45).

The primary data obtained from this study demonstrated that the therapeutic intervention attenuated lung inflammation, which correlated with negative modulation of Th17 responses in the chronic phase and with an increase in the total frequency of CD8^+^IFN-γ^+^ and γδT cells. The Th17 immune response, mediated primarily by the cytokine IL-17, is only pivotal during the early phase of TB, and an exacerbation in this response engenders progression of the pathology. It is also important to note that IFN-γ-mediated signaling is important for limiting IL-17 production and preventing the evolution of disease, demonstrating the necessary fine-tuned regulation of the immune system. This investigation highlighted the importance of investigating beyond the specific immune response to the antigen, seeking to clarify the role of other protagonists of the immune response, such as the nonconventional population of γδ T lymphocytes (45).

Once the relevance of IFN-γ for protection against TB was known, it was reasonable to evaluate one of the primary inhibitors of this cytokine: CD4^+^Foxp3^+^ regulatory cells (Treg). Investigators sought to elucidate whether there was any correlation between the protective role of DNAhsp65 during infection and the frequency of splenic and pulmonary Treg cells. Unexpectedly, under the homologous immunization with DNAhsp65, the frequency of CD4^+^Foxp3^+^ cells in the spleen was higher than that in the nonimmunized group. In contrast, heterologous immunization, which involves a prime-boost protocol where the BCG is administered to prime the cells and DNAhsp65 is administered to boost (BCG/DNAhsp65) or BCG to prime and culture filtrate proteins (CFP)–CpG to boost (BCG/CFP-CpG), reduced the frequency of Treg cells in the spleen, which led to an increase in the proportion of effector T-CD4^+^ cells per CD4^+^FoxP3^+^ cells, a ratio used as a determining factor of disease progression. In addition, BCG/DNAhsp65 vaccination improved lung preservation in comparison with the BCG/CFP-CpG, DNAhsp65 and BCG vaccinated groups (38). This analysis suggests that the prime-boost strategy is a promising alternative for inducing an effective immune response against TB (38).

Aimed at developing an effective vaccine against TB, some authors also focused on identifying the role of B lymphocytes in the protection exhibited by DNAhsp65 (46, 47). Exercising their function as APCs, B cells incorporate the plasmid DNA and modulate the cytotoxic memory response after Mtb challenge (46). The mechanisms involved in this modulation were subsequently investigated. The frequency of CD4^+^ and CD8^+^ effector memory T cells (CD44^hi^CD62L^lo^) and CD8^+^ antigen-specific memory cells (CD44^hi^CD62L^lo^CD127^+^) was monitored in wild type (WT) and B cell-deficient mice (BKO), and mRNA expression of IFN-γ, IL-12, and IL-10 was quantified in the spleen and in purified B cells by quantitative real-time PCR (RT-qPCR). Results not only suggested that IL-10-producing B cells restrain the expression of pro-inflammatory cytokines in the spleen but also led to increased survival of CD4^+^TEM cells and CD8^+^ antigen-specific memory cells after DNAhsp65 immunization. In a pro-inflammatory environment, where T cells receive numerous activation stimuli that culminate in the apoptosis of lymphocytes, B cells play a key role in immunomodulation by attenuating the highly pro-inflammatory response by the DNAhsp65 vaccination.

To better understand the immunomodulation induced by DNAhsp65, comprehensive gene expression profiling was performed in the lungs of mice infected with *M. tuberculosis* following DNAhsp65 immunotherapy (48, 49). This functional analysis of differentially expressed genes clearly discriminated the DNAhsp65 vaccinated and control groups. The DNA immunotherapy boosted the Th1 profile of response and inhibited the Th2 cytokines, regulating the amplitude of the pro-inflammatory response by the epigenetic regulation of genes related to IL-17, lymphotoxin A, TNF-α, IL-6, transforming growth factor (TGF)-β, inducible nitric oxide synthase (iNOS), and forkhead box P3 (FoxP3). In addition, the transcriptional signature observed in DNAhsp65-treated mice correlated with the attenuated injury observed in the lungs, showing larger areas of tissue preservation (48). Indeed, the data reinforce the role of HSP not only as a mycobacterial antigen but also as a peptidase, chaperone, and DC stimulator *via* TLR-4, and integration of these abilities leads to the observed immunomodulation (48). A promising application of the transcriptional results obtained would identify biomarkers of the DNA immunotherapy against TB infection (48).

Once certain of the immunotherapeutic potential of DNAhsp65, the application of this therapy in conjunction with other drugs was investigated. To that end, mice challenged with the H37Rv strain or a clinical isolated MDR-TB strain in a high bacterial burden were submitted to different protocols of therapy, including BCG, DNAhsp65, empty vector, drugs (isoniazid and pyrazinamide), or a combination of these modalities. Therapeutic intervention with DNAhsp65 and drug treatment revealed an immediate reduction in viable bacteria in the lungs in the first and third months after the injection of the first dose. Six months after the beginning of the immunotherapy, both the DNAhsp65 and the drugs diminished the colony-forming unit (CFU) count by 85 and 77%, respectively. In summary, DNAhsp65 combined with drugs was the most effective treatment for enhancing the bacterial clearance of MDR-TB infected mice compared with the other treatments, suggesting its potential for combination with conventional chemotherapeutic antibacterial drugs (39).

### Helminths–Tuberculosis Coinfection

A helminth infection causes important regulation of the immune response to Mtb antigens and negatively affects diagnosis through the Purified Protein Derivative (PPD) test. Coinfection with *M. tuberculosis* and helminths has been observed in several low-income countries and leads to different patterns of immune response. Helminth infections lead to a Th2 immune response in hosts that is characterized by IL-4 and IL-5 production, eosinophilia, and high titers of IgE in addition to regulatory cytokine production, such as TGF-β and IL-10 (50). Corroborating this, a study in Ethiopia demonstrated that peripheral blood mononuclear cells (PBMCs) from individuals with helminth infections immunized with BCG exhibited reduced IFN-γ secretion and increased TGF-β production when stimulated *in vitro* with PPD (51). In summary, helminth infections modulate the Th2 response pattern, impairing immune responses to helminth antigens and immunization efficacy, especially in individuals who are BCG immunized.

In previous work, investigators focused on the impact of the coexistence of worms and TB during immunization with DNAhsp65. They demonstrated that DNA treatment inhibited *in vivo* TGF-β and IL-10 production and increased Hsp65-specific IFN-γ secretion; moreover, bacterial burden in the lung was reduced during helminth coinfections, indicating that immunization with DNAhsp65 was efficient and persistent, even in the presence of a Th2 immune response (43). A protective role of the DNAhsp65 vaccine has been demonstrated in *Schistosoma mansoni* egg-induced pulmonary fibrosis, which causes type 2 granulomas. Immunization significantly reversed the Th1 immune response pattern by reducing CD4^+^ Th2 cell numbers, decreasing IL-4 and IL-13 cytokine production and increasing IFN-γ and IL-12 production. Therefore, the DNAhsp65 vaccine seems to efficiently target fibrotic process that arises from pathological infections due to its ability to reduce the Th2 immune response and preserve the lung parenchyma by inhibiting collagen deposition (42). Similarly, simultaneous vaccination with DNASm14 and DNAHsp65 caused an increase in TCD8^+^ cell numbers and reduced collagen deposition in the hepatic granuloma, demonstrating protection and antifibrotic effects of this combined vaccine strategy (44).

### Leishmania Infection

Other DNA vaccines expressing leishmania antigenic proteins have been developed and are potent immunity inducers, leading to protection against an experimental leishmaniasis model (52, 53). A novel formulation containing an adjuvant molecule trehalose dimycolate (TDM) and DNAhsp65 encapsulated in biodegradable microspheres of polyglycolic-colactic acid (PLGA) to provide stability and efficient delivery of these compounds has been used as an immunization strategy. The action of TDM and the DNAhsp65 vaccine in eliciting an innate immune response and specific antigen, respectively, improved the efficacy of vaccination for TB (54). Therefore, the existence of homology between leishmania and mycobacterial antigens might contribute to the success of the DNAhsp65 vaccine in *Leishmania major*-infected mice, as demonstrated by Coelho et al. (55), in which mice treated with the formulation DNAhsp65/TDM-loaded PLGA conferred protection against *L. major* infection, favoring a Th1 immune response through IFN-γ production, elevating levels of IgG2a anti-soluble *Leishmania* antigen (SLA), and decreasing levels of IL-4 and IL-10, culminating in attenuated edema and parasite numbers in the infectious site (55). In conclusion, these findings indicate that DNAhsp65 vaccines based on microspheres effectively generate protection and can be considered a new successful therapeutic approach against *Leishmania* infections. Further studies are needed to determine whether the protective role can be applied during infection with other *Leishmania* species.

### Fungal Infection

Systemic paracoccidioidomycosis is the most prevalent mycosis in Latin American countries caused by the *Paracoccidioides brasiliensis* agent. During the chronic phase, the disease affects the lungs, causing granuloma formation and a predominantly Th2-type immune response, which contributes to disease susceptibility. On the other hand, infection control is related to IFN-γ production by Th1 helper CD4^+^ cells (56). Current treatment is quite long-term, resulting in the abandonment of therapy, in addition to presenting toxicity, low efficacy, and resistance to drugs and consequently, disease relapse. Therefore, in an attempt to resolve this issue, the DNAhsp65 vaccine was studied, and its efficacy was analyzed for the treatment of fungal infections. During experimental infection with *P. brasiliensis*, DNAhsp65 immunization demonstrated prophylactic and therapeutic roles (57, 58), promoting increased nitric oxide (NO), IL-12, and IFN-γ production by splenocytes. Furthermore, increased IgG2a in serum titers was observed, and pulmonary fungal burden was consequently decreased. Moreover, equal parameters were verified during the same infection model, followed by a promising approach to delivering vaccines. In this work, the authors used liposomes or PLGA systems to deliver DNAhsp65, and both strategies promoted a Th1 immune response and reduced infection by *P. brasiliensis* (59), illustrating that liposome-based vaccines can be administered intranasally and presenting a less invasive therapeutic approach for patients. Similarly, the DNAhsp65 vaccine administered with the chemotherapeutics itraconazole and amphotericin B enhanced the efficacy of experimental chromoblastomycosis treatment (60). These results present an alternative therapy through the use of the DNAhsp65 vaccine only or associated with existing drugs due to its ability to modulate the pattern of immune response, which can reduce damage caused by fungal infections.

## DNAHSP65 in Autoimmune Diseases Modulation

Due to the homology between mammalian and mycobacterial Hsps and the ability to generate humoral and cellular immune responses in diabetes (61, 62), the possibility of generating autoimmunity after DNAhsp65 vaccine administration has become a threat to the security and applicability of this strategy. Despite this data, treatment with human HSP60 peptides (p277) or human HSP60 encoding DNA reduced the progression of insulitis and promoted the downregulation of T cell proliferation through the induction of the Th2 immune response (63). In this sense, DNAhsp65 immunization in experimental diabetes (NOD—nonobese diabetic mice and streptozotocin induced diabetes) did not trigger or accelerate development of the diabetogenic process in the pancreatic islets. In addition, the insulitis score was decreased as a result of reduced T-CD4^+^ and T-CD8^+^ cell infiltration and Treg-IL-10^+^ activation (64, 65). Moreover, when cocultures of APCs and NOD mouse T cells were incubated in the presence of DNAhsp65, Hsp65, or Hsp70 mycobacterial HSPs, the response pattern was altered from Th17 to Treg cells, showing increased IL-10 secretion and reduced IL-6, IFN-γ, and IL-17 cytokine production (66). The prime-boost strategy involving the BCG and DNAhsp565 vaccines (BCG/DNAhsp65) used as a new treatment approach reestablished blood glucose levels, reduced the inflammatory process in pancreatic islets, and promoted increased weight only in NOD mice. In contrast, this improvement was not observed in the streptozotocin model of type 1 diabetes (67). Likewise, the DNAhsp65 vaccine has also been evaluated for its ability to induce other autoimmune diseases, such as arthritis and encephalomyelitis to determine whether it has therapeutic properties in combating these diseases. Previously, the work by Rodríguez-Narciso et al. (68) showed a recovery of body weight and reduced inflammation in the joints after oral administration of recombinant HSP65 protein produced in tobacco in rat adjuvant-induced arthritis. Corroborating this data, other works found that the administration of the DNAhsp65 vaccine did not induce arthritis (69) or encephalomyelitis in animal models, even when using the BCG/DNAhsp65 prime-boost approach (70). Furthermore, immunization subsequent to disease establishment mediated a protective effect, downmodulating IL-6 and IL-12 production and upregulating levels of IL-10 in animals with induced arthritis, reducing only IL-6, TNF-α, IFN-γ, and IL-17 cytokines without changing levels of IL-10 or increasing the frequency of Tregs in the spleen of experimental autoimmune encephalomyelitis (EAE) mice (71). Likewise, when administered prior to EAE induction, the DNAhsp65 vaccine increased IL-10 production in the central nervous system (CNS) and controlled the development of EAE (72).

In summary, these data indicate that DNAhsp65 does not induce autoimmunity and is effective as a prophylactic and therapeutic in models of both autoimmune diabetes and induced arthritis, acting as a prophylactic for EAE and reinforcing its potential use as a treatment in clinical trials.

## DNAHSP65 Antitumoral Activity

After the initial efficacy results of DNAhsp65 in proof-of-concept studies on tumor immunotherapy in mice and dogs, the therapeutic properties of the DNAhsp65 vaccine were evaluated in tumors during a phase 1 clinical trial in which 21 patients with advanced head and neck squamous cell carcinoma (HNSCC) submitted to treatment. DNAhsp65 immunotherapy did not show serious side effects or autoimmune manifestations. Out of 14 patients who completed treatment, 4 of them showed partial response, and 2 are still alive more than 3 years after the end of the trial (14, 73). In addition, another work has shown that vascular endothelial growth factor (VEGF) production was reduced after immunization with DNAhsp65 in a neural TB (74) model. This may explain, in part, our results and the performance of this immunomodulator in tumor immunotherapy, suggesting that vaccination with DNA-hsp65 is a feasible and safe approach that represents a new perspective for the treatment of malignant tumors.

## Immunomodulatory Effects of DNAHSP65 in Pulmonary Diseases

### Airway Allergic Inflammation

The immune response against allergens is mediated through the action of IL-4, IL-5, and IL-13 cytokines, predominantly produced by T-helper CD4^+^ cells and by high titers of IgE. This response leads to secretion of histamine, leukotrienes, and prostaglandins by mast cells, eosinophils, and basophils. Together, cells and their mediators play an important role during allergic disorders, such as asthma, atopic dermatitis, and others, being considered potent biomarkers for prognoses and therapeutic targets. Currently, conventional therapies often require repeated and long-term doses of allergen injections; in addition to low efficacy in several allergic processes, these treatments can induce systemic reactions (75). An alternative strategy for the treatment and control of allergic disorders is through the administration of BCG (76), *Mycobacterium vaccae* (77), or microbial products as therapy, such as bacterial CpG, which inhibits the allergic process by inducing type-1 immunity (78–80). In this sense, the work by Fonseca et al. (80) demonstrated that the DNAhsp65 vaccine reduced the following parameters: eosinophilia, IgE production, pulmonary inflammation, airway hyperresponsiveness, Th2 cytokines, and mucus production in a manner dependent on IL-10 action and consequently impairing the establishment of airway allergy in mice (80). In parallel, using an ovalbumin-induced asthma experimental model, the same group demonstrated that the administration of the recombinant protein Hsp65 + CpG contributed to the activation of the innate immune response mediated by TLR9, attenuated eosinophilia, and reduced the production of Th2 cytokines. The treatment also increased levels of IFN-γ and the frequency of lung inflammatory monocytes (19). The mechanisms of action involved in these therapeutic processes are not well understood; however, it is known that DNAhsp65 stimulates MyD88 signaling, and that CpG activates Fas molecules, culminating in IFN-γ and IL-10 production and reducing allergy (81). Taken together, the DNAhsp65 vaccine or HSP65 plus CpG are promising treatments for allergic disorders and should be considered promising factors for clinical trials.

### The Fibrotic Process

Chronic pulmonary fibrosis is a disease that leads to the destruction of alveoli, irreversible loss of lung function, and high mortality rates. It is mediated by the action of innate and adaptive immune system cells, which promote the process of fibrogenesis, resulting in excessive deposition of collagen in the lung tissue. Myofibroblasts are responsive to soluble mediators (cytokines and chemokines) and initiate the fibrotic process by stimulating cytokines, such as IL-9, IL-13, IL-17, and TGF-β. In contrast, IFN-γ and IL-22 inhibit the deposition of extracellular matrix components (ECM) in these cells (82, 83). Some monoclonal antibody treatments are currently available that target profibrotic cytokines or activate Th1 cells as a novel immunopharmacological intervention (84). In this context, treatment with DNAhsp65 in an experimental model of pulmonary fibrosis reduced the deposition of noncollagenous matrix (85), and when associated with anti-TB drugs, it was more efficient in reducing tissue damage, resulting in reduced fibrosis during synergistic immunochemotherapy in experimental TB (20). Taken together, these data indicate that treatment with DNAhsp65 was effective in reducing tissue damage caused by excessive ECM deposition.

## Discussion

The DNAhsp65 vaccine has been shown to be a potential weapon for intervention in different diseases and pathological conditions. Different approaches have been used to investigate its immunobiological activities as described above, highlighting its potential as both a preventive and therapeutic intervention in a range of infectious diseases and its therapeutic applications in autoimmune diseases, allergy, and cancer. Although there is still no DNA vaccine licensed for use in humans, the licensure of four animal health products (86), including two prophylactic vaccines against infectious diseases, one gene therapy delivery of a hormone for a food animal, and one immunotherapy for cancer, provides evidence of the efficacy of DNA vaccines in multiple species.

The immunotherapeutic capacity of DNAhsp65 to control infectious diseases, autoimmune diseases, allergy, and tumors, which have significant differences in their immunopathology, is associated with a number of factors with distinct immunomodulatory capabilities inherent to the structure and/or composition of DNAhsp65 (4). The main factors that stand out include the presence of an HSP that has significant activities in the recognition, intracellular trafficking, antigen presentation (peptide), and interaction with receptors and signaling for activation of the innate and the adaptive immune system (17–21); the ability of HSPs to bind and form complexes with peptides derived from tumor or infected cells and to induce innate and/or adaptive immune response against chaperoned peptides (5, 9, 14); a DNA vaccine that by itself releases endogenous antigens and/or immunomodulators to stimulate innate and adaptive (cellular and humoral) immunity (9, 24); the presence of immunostimulatory DNA sequences containing CpG motifs in the plasmid DNA backbone that is considered to be an important adjuvant for generating the innate and Th1 immune response (17, 78–80); the double-stranded structure of the DNA plasmid that is also thought to be an immune stimulant through non-TLR mechanisms (acting on the TBK1-STING pathway through cytosolic receptors), resulting in the generation of Type-1 interferon, which then act as an adjuvant for the generation of immune responses against the antigen(s) encoded by the plasmid DNA vaccine (87, 88); the capacity for activation and regulation of adaptive immune response among Th1, Th2, Th17, Treg, and γδT pattern of lymphocytes (38, 45, 64–66, 71); and the properties of formulations or particulate delivery system and/or adjuvants to stimulate innate and adaptive immunity (27, 28, 54).

In TB, the results obtained with the DNAhsp65 vaccine containing the gene that expresses the mycobacterial heat shock protein hsp65 showed that this plasmid DNA construction is a product of radical innovation because it acts in all essential and possible points for the control of disease; acts in prevention (29–36); can be used as a BCG vaccine boost (38, 67, 70); acts in the immunotherapy of chronic TB (38), MDR-TB (39–41), and TBL (39–41); can be used concomitantly with antimycobacterial drugs to enhance the therapeutic and immunotherapeutic action (20, 39); prevents the reactivation of the infection (39, 40); and can act on those individuals with compromised immune systems, as in helminth infections (42–44).

The results of immunogenicity and therapeutic efficacy in experimental models of TB in cell culture and in animals showed that the preventive and therapeutic activities of DNAhsp65 are associated with a series of factors related to the activation of the innate and adaptive immune systems. The results of immunogenicity and efficacy tests demonstrated that the vaccine activates DCs, macrophages, and B lymphocytes; activates CD4, CD8, and γδT cells; controls the activation of lymphocytes of the Th1, Th17, and Treg patterns; guides a Th1 immune response profile, with high IFN-γ signaling; produces cytokines and activation molecules needed to control the growth of bacilli; and activates fibrosis control and resolution of the granulomatous process (20, 85). Due to the great immunotherapeutic potential of DNAhsp65 for TB, we are starting a clinical study to test the safety, maximum tolerated dose, immunogenicity, and preliminary efficacy of this product as a therapeutic adjunct for the treatment of patients with MDR-TB (DNAhsp65 + drugs).

Studies with DNAhsp65 have not only made clear the immunomodulatory role of this product against TB but have shown efficient immunotherapeutic activities for the control of other infectious diseases where the immune system has a primary role, such as leishmaniasis (55), paracoccidioidomycosis (57–59), schistosomiasis (42–44), and chromoblastomycosis (60). As in TB, helminths, leishmania, and fungal infections, DNAhsp65 exhibits consolidated properties to counterbalance the immune response from Th2 to Th1, with augmented expression of IFN-γ and IL-12.

Because the DNAhsp65 vaccine biases the T-helper cell response to a Th1 phenotype, the DNAhsp65 vaccine is also under development as an immunotherapy against allergy. Using an experimental model of airway allergic inflammation in mice, DNAhsp65 immunotherapy attenuated eosinophilia, IgE production, pulmonary inflammation, airway hyperresponsiveness, Th2 cytokines, and mucus production (80). Cells transferred from DNAhsp65-immunized mice to allergic mice migrated to allergic sites and downregulated the Th2 response. These findings clearly show that immunotherapy with DNA encoding Hsp65 attenuates an established Th2 allergic inflammation through an IL-10-dependent mechanism (80). We also investigated the participation of the innate response, particularly the role of the MyD88 adaptor, and Fas molecules in the effectiveness of DNAhsp65 or CpG/culture filtrated proteins (CFP) immunotherapy. Notably, transfer of cells from DNAhsp65- or CpG/CFP immunized MyD88^−/−^ knockout mice failed to reduce allergy (81). Additionally, for effective reduction of allergy by cells from DNAhsp65-immunized mice, Fas molecules were required (81). In addition to immune redirection to a Th1 response in the modulation of Th2 allergic inflammation, our findings also attribute an important role to the innate response mediated by TLR9, which is associated with the recruitment of CCR2-dependent monocytes (19).

In addition to the finding described above, in airway allergic inflammation and fibrotic processes, the DNAhsp65 diminished the Th2 response and IL-4 and IL-5 cytokine releases, leading to increased expression of IFN- and IL-22, which is related to the inhibition of inflammatory monocytes in allergy and decreases the deposition of ECM in fibrotic process. Despite this, the DNAhsp65 application in health programs still requires advanced human or animal studies and clinical trials to amplify knowledge on its biological safety, dosage, administration routes, and possible side effects in each of these contexts. Some studies are still under development using the DNAhsp65 vaccine as a treatment for atopic dermatitis in dogs.

The mechanism involved in the control of autoimmune diseases also involves the immunoregulatory property of elevating IL-10 expression and Treg signaling, attenuating the self-reactive T lymphocyte responses.

The ability of DNAhsp65 to participate in both innate and adaptive immune responses also makes it a promising candidate for cancer immunotherapy. In a proof of concept study in mice, the antitumoral activity of DNAhsp65 was correlated with increased percentages of activated lymphocyte (CD4/CD44^hi^, CD8/CD44^hi^) infiltration into the tumor mass, enhanced CD86 costimulatory molecule expression in APCs and CD8-T lymphocytes specific lysis. Furthermore, gene expression in tumors was modulated by DNA-Hsp65 intratumoral treatment, leading to increased T-bet and IL-17 gene expression.

Considering that DNAhsp65 elicits antitumoral effects, we proposed a Phase I/II trial of DNAhsp65 immunotherapy for advanced HNSCC (14). DNAhsp65 immunotherapy is a feasible and safe approach at a dose of 400 mg per injection in patients with HNSCC refractory to standard treatment. Immunological evaluation of patients in this clinical trial included a humoral response to mycobacterial Hsp65 and human Hsp60, antigen-specific proliferation, and both IFN-γ and IL-10 production by ELISPOT assay. Of note, in the present report, almost all patients (19 out of 21) showed signs of immune stimulation, and 4 patients exhibited a decrease in tumor size as well, suggesting that the vaccine interferes with the immunological status of patients. Moreover, in four patients, spontaneous PBMC proliferation increased after vaccination, and in two patients, a progressive increase was detected during the course of treatment (73). These results, together with data already published about the clinical trial for patients with cervical tumors, will be of great importance to highlight the DNAhsp65 vaccine as a promising immunotherapy in malignant cancer.

In summary, this review described the state of the art research on the DNAhsp65 vaccine, highlighting its property as an intrinsic mycobacterial antigen, its chaperone and adjuvant capacity due to the role played by hsp65, its preventive and immunotherapeutic uses in different aspects of TB, its potential to control infectious conditions and autoimmune diseases, its immunoregulation of antitumoral responses, and its immunomodulatory property of switching the immune response toward the Th1 profile. Taken together, these findings indicate that the DNAhsp65 vaccine represents a strong candidate for the treatment of diseases contextualized in this review.

## Author Contributions

TM and AT wrote the sessions of this review. CS reviewed all written sessions and contributed to enrich the discussion. All authors contributed to the article and approved the submitted version.

## Conflict of Interest

The authors declare that the research was conducted in the absence of any commercial or financial relationships that could be construed as a potential conflict of interest.

## References

[1] Riedel S. Edward Jenner and the History of Smallpox and Vaccination. Baylor University Medical Center Proceedings. (2005) p. 21–25. 10.1080/08998280.2005.11928028 PMC1200696 16200144

[2] Paoletti E Lipinskas BR Samsonoff C Mercer Panicali SD. Construction of live vaccines using genetically engineered poxviruses: biological activity of vaccinia virus recombinants expressing the hepatitis B virus surface antigen the herpes simplex virus glycoprotein D. Proc Natl Acad Sci USA. (1984) 81:193–7. 10.1073/pnas.81.1.193 6320164 PMC344637

[3] Gómez A Oñate LA. A Plasmid-Based DNA Vaccines, Plasmid, Munazza Gull. IntechOpen. (2018) 10.5772/intechopen.76754

[4] Silva CL. “Gene Therapy” in Animal Cell Technology: From Biopharmaceuticals to Gene Therapy. Taylor & Francis Group. (2008). p. 489–518.

[5] Binder RJ. Functions of heat shock proteins in pathways of the innate and adaptive immune system. J Immunol. (2014) 193:5765–71. 10.4049/jimmunol.1401417 25480955 PMC4304677

[6] Lindquist S. The heat-shock response. Annu Rev Biochem. (1986) 55:1151–91. 10.1146/annurev.bi.55.070186.005443 2427013

[7] Srivastava P. Roles of heat-shock proteins in innate and adaptive immunity. Nat Rev. Immunol. (2002) 2:185–94. 10.1038/nri749 11913069

[8] Binder PK Srivastava RJ. Peptides chaperoned by heat shock proteins are a necessary and sufficient source of antigen in the cross-priming of CD8+ T cells. Nat Immunol;. (2005) 6:593–9. 10.1038/ni1201 15864309

[9] Quintana FJ Cohen IR. Heat shock proteins as endogenous adjuvants in sterile and septic inflammation. J Immunol. (2005) 175:2777–82. 10.4049/jimmunol.175.5.2777 16116161

[10] Van Eden W van der Zee R Prakken B. Heat-shock proteins induce T-cell regulation of chronic inflammation. Nat Rev Immunol. (2005) 5:318–30. 10.1038/nri1593 15803151

[11] Quintana FJ Cohen IR. DNA vaccines coding for heat shock proteins (HSPs). Tools for the activation of HSP-specific regulatory T cells. Expert Opin Biol Ther. (2005) 5:545–54. 10.1517/14712598.5.4.545 15934832

[12] Binder RJ. Heat shock protein vaccines: from bench to bedside. Int Rev Immunol. (2006) 25:353–75. 10.1080/08830180600992480 17169780

[13] Souza CL Silva AO. Vaccines of the future: from rational design to clinical development. Exp Opin Biol Ther. (2002) 2:219–22. 10.1517/14712598.2.2.219 12170946

[14] Michaluart P Abdallah KA Lima FD Smith R Moysés RA Coelho V. Phase I trial of DNA-hsp65 immunotherapy for advanced squamous cell carcinoma of the head and neck. Cancer Gene Therapy. (2002) 5:676–84. 10.1038/cgt.2008.35 18535616

[15] Portaro Hayashi FCV MAF Arauz LJ Palma MS Silva CL. The hsp65 displays proteolytic activity. Mutagenesis studies indicate that the hsp65 proteolytic activity is catalytically related to the HslVU protease. Biochemistry. (2002) 41:7400–6. 10.1021/bi011999l 12044173

[16] Rossetti RAM Lorenzi JCC Giuliatti S Silva CL Coelho-Castelo AAM. *In silico* prediction of the tertiary structure of, *M. leprae* Hsp65 protein shows an unusual structure in carboxi-terminal region. J Comp Sci Syst Biol. (2008) 1:126–31. 10.4172/jcsb.1000012

[17] Franco LH Wowk PL Silva CL Trombone AAM Coelho-Castelo APF. A DNA vaccine against tuberculosis based on the 65 kDa heat-shock protein differentially activates human macrophages and dendritic cells. Genet Vacc Ther. (2008) 6:1–11. 10.1186/1479-0556-6-3 18208592 PMC2267464

[18] Wowk PF Franco LH Fonseca DM Paula MO Viana ESO Wedling P. Mycobacterial Hsp65 antigen upregulates the cellular immune response of healthy individuals compared to tuberculosis patients. Hum Vacc Immunother. (2017) 13:1040–50. 10.1080/21645515.2016.1264547 28059670 PMC5443371

[19] Prado Bertolini RQ TB Piñeros AR Gembre AF Ramos SG Silva L. Attenuation of experimental asthma by mycobacterial protein combined with CpG requires a TLR9-dependent IFN-γ-CCR2 signaling circuit. Clin Exp Aller. (2015) 45:1459–71. 10.1111/cea.12564 25944185

[20] Rodrigues RF Zárate-Bladés CR Rios WM Soares LS Souza ZMPR. Synergy of chemotherapy and immunotherapy revealed by a genome-scale analysis of murine tuberculosis. J Anti Chem. (2015) 70:1774–83. 10.1093/jac/dkv023 25687643

[21] Trombone APF Silva CL Almeida LP Rosada RS Lima KM Oliver M. Tissue distribution of DNA-Hsp65/TDM-loaded PLGA microspheres and uptake by phagocytic cells. Gen Vacc Ther. (2007) 5:1–8. 10.1186/1479-0556-5-9 17880727 PMC2042973

[22] Kim TH Shin SJ Park YM Jung ID Ryu SW Kim DJ. Critical role of TRIF and MyD88 in Mycobacterium tuberculosis Hsp70-mediated activation of dendritic cells. Cytokine. (2015) 71:139–44. 10.1016/j.cyto.2014.09.010 25461391

[23] Palliser D Huang Q Hacohen N Lamontagne SP Guillen E Young RA. A role for toll-like receptor 4 in dendritic cell activation and cytolytic CD8+ T cell differentiation in response to a recombinant heat shock fusion protein. J Immunol. (2004) 172:2885–93. 10.4049/jimmunol.172.5.2885 14978090

[24] Vabulas RM Ahmad-Nejad P Ghose S Kirschning CJ Issels RD Wagner H. HSP70 as endogenous stimulus of the Toll/interleukin-1 receptor signal pathway. J Biol Chem. (2002) 277:15107–2. 10.1074/jbc.M111204200 11842086

[25] Qazi KR Oehlmann W Singh M Lopez MC Fernandez C. Microbial heat shock protein 70 stimulatory properties have different TLR requirements. Vaccine. (2007) 25:1096–3. 10.1016/j.vaccine.2006.09.058 17049413

[26] Asea A Rehli M Kabingu E Boch JA Bare O Auron PE. Novel signal transduction pathway utilized by extracellular HSP70: role of toll-like receptor (TLR) 2 and TLR4. J Biol Chem. (2002) 277:15028–34. 10.1074/jbc.M200497200 11836257

[27] Souza PRM Zárate-Bladés CR Hori JI Ramos SG Lima DS Schneider S. Protective efficacy of different strategies employing *Mycobacterium leprae* heat-shock protein 65 against tuberculosis. Expert Opin Biological Ther. (2008) 8:1255–64. 10.1517/14712598.8.9.1255 18694348

[28] Lima KM Santos SA Junior JMR Silva CL. Vaccine adjuvant: it makes the difference. Vaccine. (2004) 22:2374–9. 10.1016/j.vaccine.2003.12.030 15193397

[29] Lowrie DB Tascon RE Colston CL Silva MJ. Towards a DNA vaccine against tuberculosis. Vaccine. (1994) 12:1537–40. 10.1016/0264-410X(94)90080-9 7879421

[30] Silva CL Bonato VLD Lima VMF. DNA encoding individual mycobacterial antigens protects mice against tuberculosis. Brazil J Med Biol Res. (1999) 32:231–4. 10.1590/S0100-879X1999000200012 10347759

[31] Lowrie DB Silva RE Tascon CL. Progress towards a new tuberculosis vaccine. Bio Drugs. (1998) 10:201–13. 10.2165/00063030-199810030-00004 18020596

[32] Lowrie DB Silva RE Tascon CL. DNA vaccines against tuberculosis. Immunol Cell Biol. (1997) 75:591–4. 10.1038/icb.1997.93 9492198

[33] Lowrie DB Silva RE Tascon CL. Genetic vaccination against tuberculosis. Sem Immunopathol. (1997) 19:161–73. 10.1007/BF00870266 9406344

[34] Lowrie DB Silva CL Colston MJ Ragno RE Tascon S. Protection against tuberculosis by a plasmid DNA vaccine. Vaccine. (1997) 15:834–8. 10.1016/S0264-410X(97)00073-X 9234527

[35] Lowrie DB Tascon CL Silva RE. Vaccination against tuberculosis. Int Arch Aller Immunol. (1995) 108:309–12. 10.1159/000237172 7580299

[36] Silva CL. New vaccines against tuberculosis. Brazil J Med Biol Res. (1995) 28:843–51. 8555985

[37] Fedato PF Sergio CA Paula MO Gembre AP Franco LH Wowk P. Protection conferred by heterologous vaccination against tuberculosis is dependent on the ratio of CD4 /CD4 Foxp3 cells. Immunology. (2012) 137:239–48. 10.1111/imm.12006 22891805 PMC3482681

[38] Silva CL Bonato VLD Lima VMF Faccioli SC Leão LH. Characterization of the memory/activated T cells that mediate the long-lived host response against tuberculosis after bacillus Calmette-Guerin or DNA vaccination. Immunology. (1999) 97:573–81. 10.1046/j.1365-2567.1999.00840.x 10457209 PMC2326889

[39] Silva CL Bonato VLD Coelho-Castelo AAM Souza AO Santos SA Lima M. Immunotherapy with plasmid DNA encoding mycobacterial hsp65 in association with chemotherapy is a more rapid and efficient form of treatment for tuberculosis in mice. Gene Ther. (2004) 12:281–7. 10.1038/sj.gt.3302418 15526006

[40] Lowrie DB Tascon RE Bonato VDL Lima VM Faccioli LH Stravopoulos H. Therapy of tuberculosis in mice by DNA vaccination. Nature. (1999) 400:269–71. 10.1038/22326 10421369

[41] Lowrie CL Silva DB. Enhancement of immunocompetence in tuberculosis by DNA vaccination. Vaccine. (2000) 18:1712–6. 10.1016/S0264-410X(99)00512-5 10689154

[42] Frantz FG Ito T Cavassani KA Hogaboam CM Silva CL Kinkel L. Therapeutic DNA vaccine reduces *Schistosoma mansoni* induced tissue damage through cytokine balance and decreased migration of myofibroblasts. Am J Pathol. (2011) 179:223–9. 10.1016/j.ajpath.2011.03.012 21703404 PMC3123877

[43] Frantz FG Rosada RS Peres-Buzalaf C Perusso FRT Rodrigues V Ramos SG. Helminth coinfection does not affect therapeutic effect of a DNA vaccine in mice harboring tuberculosis. Plos Negl Trop Dis. (2010) 4:e700. 10.1371/journal.pntd.0000700 20544012 PMC2882318

[44] Espíndola MS Frantz FG Soares SL Masson AP Tefé-Silva C Bitencourt S. Combined immunization using DNA-Sm14 and DNA-Hsp65 increases CD8+ memory T cells, reduces chronic pathology and decreases egg viability during *Schistosoma mansoni* infection. BMC Infect Dis. (2014) 14:263. 10.1186/1471-2334-14-263 24886395 PMC4031977

[45] Zarate-Bladés CR Rodrigues RF Souza PRM Rios WM Soares LS Rosada S. Evaluation of the overall IFN-γ and IL-17 pro-inflammatory responses after DNA therapy of tuberculosis. Hum Vacc Immunother. (2013) 9:13–23. 10.4161/hv.23417 23324590 PMC3899145

[46] Fontoura IC Trombone APF Almeida LP Lorenzi JCC Rossetti AMR Malardo T. B cells expressing IL-10 mRNA modulate memory T cells after DNA-Hsp65 immunization. Brazil J Med Biol Res. (2015) 48:1095–100. 10.1590/1414-431x20154409 26397973 PMC4661025

[47] Almeida LP Trombone APF Lorenzi JCC Rocha CD Malardo T Fontoura C. B cells can modulate the CD8 memory T cell after DNA vaccination against experimental tuberculosis. Gen Vacc Ther. (2011) 9:5. 10.1186/1479-0556-9-5 21401938 PMC3066104

[48] Zárate-Bladés CR Bonato VLD Silveira ELV Paula MO Junta CM Sandrin-Garcia C. Comprehensive gene expression profiling in lungs of mice infected with Mycobacterium tuberculosis following DNAhsp65 immunotherapy. J Gene Med. (2009) 11:66–78. 10.1002/jgm.1269 19035575

[49] Zárate-Bladés CR Silva GA Passos CL. The impact of transcriptomics on the fight against tuberculosis: focus on biomarkers, BCG vaccination, and immunotherapy. Clin Dev Immunol. (2011) 2011:1-6. 10.1155/2011/192630 21197423 PMC3010624

[50] Maizels RM Yazdanbakhsh M. Immune regulation by helminth parasites: cellular and molecular mechanisms. Nat Rev Immunol. (2003) 3:733–44. 10.1038/nri1183 12949497

[51] Elias D Britton S Aseffa A Engers H Akuffo H. Poor immunogenicity of BCG in helminth infected population is associated with increased *in vitro* TGF-beta production. Vaccine. (2008) 26:3897–902. 10.1016/j.vaccine.2008.04.083 18554755

[52] Ghosh A Labrecque S Matlashewski G. Protection against *Leishmania donovani* infection by DNA vaccination: increased DNA vaccination efficiency through inhibiting the cellular p53 response. Vaccine. (2001) 19:3169–78. 10.1016/S0264-410X(01)00023-8 11312013

[53] Ahmed SBH Bahloul C Robbana C Askri S Dellagi K. A comparative evaluation of different DNA vaccine candidates against experimental murine leishmaniasis due to, *L. major*. Vaccine. (2004) 22:1631–9. 10.1016/j.vaccine.2003.10.046 15068845

[54] Lima KM Santos SA Lima VMF Coelho-Castelo AAM Rodrigues JM Silva CL. Single dose of a vaccine based on DNA encoding mycobacterial hsp65 protein plus TDM-loaded PLGA microspheres protects mice against a virulent strain of *Mycobacterium tuberculosis*. Gene Ther. (2003) 10:678–85. 10.1038/sj.gt.3301908 12692596

[55] Coelho EAF Tavares CAP de Melo Lima K Silva CL Rodrigues Jr JM Fernandes AP. Mycobacterium hsp65 DNA entrapped into TDM-loaded PLGA microspheres induces protection in mice against *Leishmania major* infection. Parasitol Res. (2005) 98:568-75. 10.1007/s00436-005-0088-5 16432754

[56] Benard G. An overview of the immunopathology of human paracoccidioidomycosis. Mycopathologia. (2008) 165:209–21. 10.1007/s11046-007-9065-0 18777630

[57] Ribeiro AM Bocca AL Amaral AC Faccioli LH Galetti FCS Zárate-Bladés CR. DNAhsp65 vaccination induces protection in mice against Paracoccidioides brasiliensis infection. Vaccine. (2009) 27:606–13. 10.1016/j.vaccine.2008.10.022 19028537

[58] Ribeiro AM Bocca AL Amaral AC Souza A Faccioli LH. HSP65 DNA as therapeutic strategy to treat experimental paracoccidioidomycosis. Vaccine. (2010) 28:1528–34. 10.1016/j.vaccine.2009.11.062 20045500

[59] Ribeiro AM Souza ACO Amaral AC Vasconcelos NM Jerônimo MS Carneiro FP. Nanobiotechnological approaches to delivery of DNA vaccine against fungal infection. J Biomed Nanotechnol. (2013) 9:221–30. 10.1166/jbn.2013.1491 23627048

[60] Siqueira IM Ribeiro AM de Medeiros Nóbrega YK Simon KS Souza ACO Jerônimo MS. DNA-hsp65 vaccine as therapeutic strategy to treat experimental chromoblastomycosis caused by fonsecaea pedrosoi. Mycopathologia. (2012) 175:463–75. 10.1007/s11046-012-9599-7 23179449

[61] Bras A Aguas AP. Diabetes-prone NOD mice are resistant to *Mycobacterium avium* and the infection prevents autoimmune disease. Immunology. (1996) 89:20–5. 10.1046/j.1365-2567.1996.d01-717.x 8911135 PMC1456667

[62] Gupta MM Raghunath D Kher SK Radhakrishnan AP. Human leucocyte antigen and insulin dependent diabetes mellitus. J Assoc Phys. (1991) 39:540–3. 1724775

[63] Ablamunits V Elias D Reshef T Cohen IR. Treatment of autoimmune diabetes and insulitis in NOD mice with heat shock protein 60 peptide p277. J Autoimmun. (1998) 11:73–81. 10.1006/jaut.1997.0177 9480725

[64] Rodrigues dos Santos R Sartori A Deperon Bonato VL Coelho Castelo AAM Vilella CA Zollner RL. Immune modulation induced by tuberculosis DNA vaccine protects non-obese diabetic mice from diabetes progression. Clin Exp Immunol. (2007) 149:570–8. 10.1111/j.1365-2249.2007.03433.x 17590177 PMC2219319

[65] Santos RR Sartori A Lima DS Souza PR Coelho-Castelo AA Bonato VL. DNA vaccine containing the mycobacterial hsp65 gene prevented insulitis in MLD-STZ diabetes. J Immun Based Ther Vacc. (2009) 7:4. 10.1186/1476-8518-7-4 19754943 PMC2754477

[66] Pileggi GS Clemencio AD Malardo T Antonini SR Bonato VLD Rios WM. New strategy for testing efficacy of immunotherapeutic compounds for diabetes *in vitro*. BMC Biotechnol. (2016) 16:1. 10.1186/s12896-016-0270-0 27165305 PMC4862051

[67] Da Rosa LC Chiuso-Minicucci F Zorzella-Pezavento SFG França TGD Ishikawa LLW Colavite PM. Bacille Calmette-Guérin/DNAhsp65 prime-boost is protective against diabetes in non-obese diabetic mice but not in the streptozotocin model of type 1 diabetes. Clin Exp Immunol. (2013) 173:430–7. 10.1111/cei.12140 23692306 PMC3949630

[68] Rodríguez-Narciso C Pérez-Tapia M Rangel-Cano RM Silva CL Meckes-Fisher M Salgado-Garciglia R. Expression of *Mycobacterium leprae* HSP65 in tobacco and its effectiveness as an oral treatment in adjuvant-induced arthritis. Trans Res. (2010) 20:221–9. 10.1007/s11248-010-9404-7 20526808

[69] Santos-Junior RR Sartori A Franco MD Filho OGR Coelho-Castelo AAM Bonato VLD. Immunomodulation and protection induced by DNA-hsp65 vaccination in an animal model of arthritis. Hum Gene Ther. (2005) 16:1338–45. 10.1089/hum.2005.16.1338 16259568

[70] Zorzella-Pezavento SFG Guerino CPF Chiuso-Minicucci F França TGD Ishikawa LLW Masson AP. BCG and BCG/DNAhsp65 vaccinations promote protective effects without deleterious consequences for experimental autoimmune encephalomyelitis. Clin Dev Immunol. (2013) 2013:1–9. 10.1155/2013/721383 24288555 PMC3830802

[71] Zorzella-Pezavento SFG Chiuso-Minicucci F França TGD Ishikawa LLW da Rosa LC Colavite PM. Downmodulation of peripheral MOG-specific immunity by pVAXhsp65 treatment during EAE does not reach the CNS. J Neuroimmunol. (2014) 268:35–42. 10.1016/j.jneuroim.2013.12.015 24439542

[72] Zorzella-Pezavento SFG Chiuso-Minicucci F França TGD Ishikawa LLW da Rosa LC Colavite PM. pVAXhsp65 vaccination primes for high IL-10 production and decreases experimental encephalomyelitis severity. J Immunol Res. (2017) 2017:1–11. 10.1155/2017/6257958 28321419 PMC5339488

[73] Victora GD Socorro-Silva A Volsi EC Abdallah K Lima FD Smith RB. Immune response to vaccination with DNA-hsp65 in a phase I clinical trial with head and neck cancer patients. Cancer Gene Ther. (2009) 16:598–608. 10.1038/cgt.2009.9 19197326

[74] Zucchi FCR Tsanaclis AMC Moura-Dias Q Silva CL Pelegrini-da-Silva A Neder L. Modulation of angiogenic factor VEGF by DNA-hsp65 vaccination in a murine CNS tuberculosis model. Tuberculosis. (2013) 93:373–80. 10.1016/j.tube.2013.02.002 23491717

[75] Manka LA Wechsler ME. New biologics for allergic diseases. Exp Rev Clin Immunol. (2018) 14:285–96. 10.1080/1744666X.2018.1459188 29611440

[76] Erb KJ Holloway JW Sobeck A Moll H Le Gros. G. Infection of mice with *Mycobacterium bovis*-Bacillus Calmette-Guérin (BCG) suppresses allergen-induced airway eosinophilia. J Exp Med. (1998) 187:561–9. 10.1084/jem.187.4.561 9463406 PMC2212158

[77] Zuany-Amorim C Sawicka E Manlius C Le Moine A Brunet LR Kemeny DM. Suppression of airway eosinophilia by killed *Mycobacterium vaccae*-induced allergen-specific regulatory T-cells. Nat Med. (2002) 8:625–9. 10.1038/nm0602-625 12042815

[78] Horner AA Redecke V Raz E. Toll-like receptor ligands: hygiene, atopy and therapeutic implications. Curr Opin Allerg Clin Immunol. (2004) 4:555–61. 10.1097/00130832-200412000-00014 15640699

[79] Rook GAW Adams V Palmer R Brunet LR Hunt J Martinelli R. Mycobacteria and other environmental organisms as immunomodulators for immunoregulatory disorders. Semin Immunopathol. (2004) 25:237–55. 10.1007/s00281-003-0148-9 15007629

[80] Fonseca DM Wowk PF Paula MO Campos LW Gembre AF Turato WM. Recombinant DNA immunotherapy ameliorate established airway allergy in a IL-10 dependent pathway. Clin Exp Allergy. (2011) 42:131–43. 10.1111/j.1365-2222.2011.03845.x 22093133

[81] Fonseca DM Wowk PF Paula MO Gembre AF Baruffi MD Fermino ML. Requirement of MyD88 and Fas pathways for the efficacy of allergen-free immunotherapy. Allergy. (2014) 70:275–84. 10.1111/all.12555 25477068

[82] Fernandez IE Eickelberg O. New cellular and molecular mechanisms of lung injury and fibrosis in idiopathic pulmonary fibrosis. Lancet. (2012) 380:680–8. 10.1016/S0140-6736(12)61144-1 22901889

[83] Wynn TA. Integrating mechanisms of pulmonary fibrosis. J Exp Med. (2011) 208:1339–50. 10.1084/jem.20110551 21727191 PMC3136685

[84] Kolahian S Fernandez IE Eickelberg O Hartl D. Immune mechanisms in pulmonary fibrosis. Am J Resp Cell Mol Biol. (2016) 55:309–22. 10.1165/rcmb.2016-0121TR 27149613

[85] Pádua AI. Influence of a DNA-hsp65 vaccine on bleomycin-induced lung injury. J Brasil Pneumol. (2008) 34:891-899. 10.1590/S1806-37132008001100002 19099094

[86] Redding DB Weiner L. DNA vaccines in veterinary use. Expert Rev Vaccines. (2009) 8:1251–76. 10.1586/erv.09.77 19722897 PMC4425421

[87] Coban C Kobiyama K Jounai N Tozuka M Ishii KJ. DNA vaccines-A simple DNA sensing matter? Hum Vaccin Immunother. (2013) 9:2216–21. 10.4161/hv.25893 23912600 PMC3906407

[88] Allen A Wang C Caproni LJ Sugiyarto G Harden E Douglas R. Linear doggybone DNA vaccine induces similar immunological responses to conventional plasmid DNA independently of immune recognition by TLR9 in a pre-clinical model. Cancer Immunol Immunother. (2018) 67:627–8. 10.1007/s00262-017-2111-y 29330557 PMC5860099

